# Oral galvanism related to dental implants

**DOI:** 10.1186/s40902-023-00403-8

**Published:** 2023-10-06

**Authors:** Soung Min Kim

**Affiliations:** 1 Department of Oral and Maxillofacial Surgery, Dental Research Institute, School of Dentistry, Seoul National University, 101 Daehak-ro, Jongno-gu, Seoul, 03080 Korea; 2 Oral and Maxillofacial Microvascular Reconstruction LAB, Ghana Health Service, Brong Ahafo Regional Hospital, P.O. Box 27, Sunyani, Brong Ahafo Ghana

**Keywords:** Oral galvanism, Galvanic corrosion, Crevice corrosion, Dental implant, Precancerous lesion with oral galvanism, Peri-implant oral malignancy

## Abstract

**Background:**

A range of different chemical interactions can generate an unexpected electronic current in a process called galvanism. Oral galvanism (OG) can also be generated by different chemical actions from diverse intraoral rehabilitated metals, including gold, copper, mercury, titanium, and titanium alloy. The main aim of this manuscript is to review OG, particularly focusing on titanium implants and related metallic materials. We searched the MEDLINE (PubMed), Embase, Scopus, and Google Scholar databases for relevant literature published through December 2019. The keywords included “galvanic current”, “galvanism”, “galvanic corrosion”, “oral galvanism”, combined with “oral”, “oral cavity”, “implant”, and “saliva.”

**Results:**

Out of 343 articles, 126 articles that met the inclusion criteria were reviewed. We examined and summarized research on OG through a division into four categories: definition and symptoms, diagnosis with testing methods, galvanic corrosion, and oral precancerous lesions with OG.

**Conclusions:**

Patients with OG have high oral energy and current, and although this phenomenon may be due to the patient’s mental illness, OG due to amalgam or mercury occurs. It is evident that the difference in electron potential caused by different elemental components such as titanium alloy and pure titanium, which are essential for manufacturing the implant fixture and the abutment, and chrome and nickel, which are essential for manufacturing the upper crown, causes OG. Since the oral cavity is equipped with an environment in which electric current can be transmitted easily due to saliva, it is imperative that clinicians review the systemic and local effects of salivation.

## Background

Oral galvanism (OG) was first reported and described as the corrosive products caused by metallic dental filling materials by Sulzer et al. in 1754 [1], and the representative amalgam filling materials were labeled as materials dangerous to health in 1870 due to their oral electricity properties [2]. Metallic biomaterials undergo chemical reactions in the oral environment to produce corrosion products. The disintegration of dental alloys may occur at a wide range of pHs and fluctuations in temperature of the oral cavity. Titanium (Ti) is a popular material in dentistry because of its chemical and mechanical stability with very low toxicity. Among the main characteristics of Ti, good biocompatibility and high resistance to corrosion in the diverse oral cavity are especially important [3, 4]. This biocompatibility and anticorrosion characteristics of titanium depend on the composition of Ti alloy and metallurgical and saliva parameters.

In the oral cavity, galvanic current is also known as galvanic corrosion (GC), which is an electrochemical response between different metal fillings and crown materials [5]. GC is found commonly in dental-implant-related restorations [6], due to direct contact between dissimilar metals. These complicated electrochemical processes related to implant and suprastructure might lead to soft tissue effects including peri-implantitis and hard tissue effects including alveolar bone destruction, due to the galvanic currents from dissoluted components of dissimilar metal alloys [6].

Tissue reactions to titanium particles have been reported to be very diverse, ranging from transient to a mild or even severe response [7]. On the other hand, in vivo evidence of the effects of galvanic current on oral tissues is now well known. Several galvanic situations in the oral cavity might influence the basic immune defensive functions, and subsequently cause oral discomfort, including galvanic lichenoid reactions [8–12]. These lichenoid reactions could be a lead to oral squamous cell carcinoma (OSCC) of the gingiva and tongue through oral precancerous cellular changes caused by OG [11–13].

Until now, there has been no comprehensive review of the literature on the effects of galvanic currents resulting from dissolved components of dissimilar metal alloys on surrounding soft tissue. The aim of this manuscript is to investigate OG related to dental implants, focusing on Ti implants and related metallic materials. This review paper is divided into four sections: definition and symptoms, diagnosis with testing methods, galvanic corrosion, and oral precancerous lesions with galvanism.

## Methods

We have reviewed under the Preferred Reporting Items for Systematic Reviews and Meta-Analyses (PRISMA) checklist as below.

Focus question is as follows: “What are OG and its association with titanium implants?”.

### Search strategy

We searched the MEDLINE (PubMed), Embase, Scopus, and Google Scholar databases for relevant literature published through December 2019. The keywords included “galvanic current”, “galvanism”, “galvanic corrosion”, “oral galvanism”, combined with “oral”, “oral cavity”, “implant”, and “saliva”. The full-term research strategy is presented in Table 1. We also checked the reference lists of included papers and collected review articles through the search for additional studies that might have been missed by the search strategy. A total of 343 articles were identified.

**Table 1:** Search term strategy of this study

Database name	Date of search	Searching string	Results
Scopus	24th, December, 2022	( ( "galvanic current" OR "galvanism" OR "galvanic corrosion" OR "oral galvanism" OR “electrogalvanic”) AND ( "oral" OR "dental") AND ( "implant" OR "saliva"))	95
PubMed	20th, December, 2022	((((("galvanic current"[All Fields] OR "galvanism"[All Fields]) OR "galvanic corrosion"[All Fields]) OR "oral galvanism"[All Fields]) OR "electrogalvanic"[All Fields]) AND ("oral"[All Fields] OR "dental"[All Fields])) AND (("implant"[All Fields] OR "saliva"[All Fields]) OR "alloy"[All Fields])	70
Embase	22th, December, 2022	('galvanic current'/exp OR 'galvanic current' OR galvanism* OR 'galvanic corrosion' OR 'electrogalvanic') AND (oral* OR dental*) AND (implant* OR saliva*)	67
Google Scholar	24th, December, 2022	( ( "galvanic current" OR "galvanism" OR "galvanic corrosion" OR "oral galvanism" OR “electrogalvanic”) AND ( "oral" OR "dental") AND ( "implant" OR "saliva"))	First 80 results^*^
Hand searching	24th, December, 2022	Reference lists of the included papers and review articles	31
Total			343

^*^A 4.140 results were produced from this search string, however; only the first 80 results from the Google Scholar database were considered and reviewed

### Inclusion criteria

Records are included if they are peer-reviewed journal or conference papers in English. Below contents are all included.

1. Describes the definition and symptoms of OG. Describes the oral precancerous lesions, such as leukoedema, leukoplakia, or oral lichen planus (OLP), relating to galvanic current. Describes the response of oral mucosa to contact with galvanic current. Describes the clinical diagnosis with testing methods for OG including the following:
  Any clinical case report or case series report
  Any “in-field” (non-lab) testing method or device to detect the galvanic current in patients
  At least one clinical evaluation metric, such as symptoms, testing, or others, for detection of galvanic current
2. Report the corrosion of galvanic pairs of dental alloys under different saliva conditions, especially the galvanism behavior between Ti implants and dental alloys of implant superstructure including the following:
  Any electrochemical techniques to measure the potential, polarization, and galvanic current
  Any tomography method to observe the corrosion effects on metal surfaces
  Any method to evaluate the composition and microstructure of particles and ion release caused by galvanic current

### Exclusion criteria

Non-English language, reviews, and commentary articles were excluded, as were duplicates.

### Screening and selection strategy

**Fig. 1:**
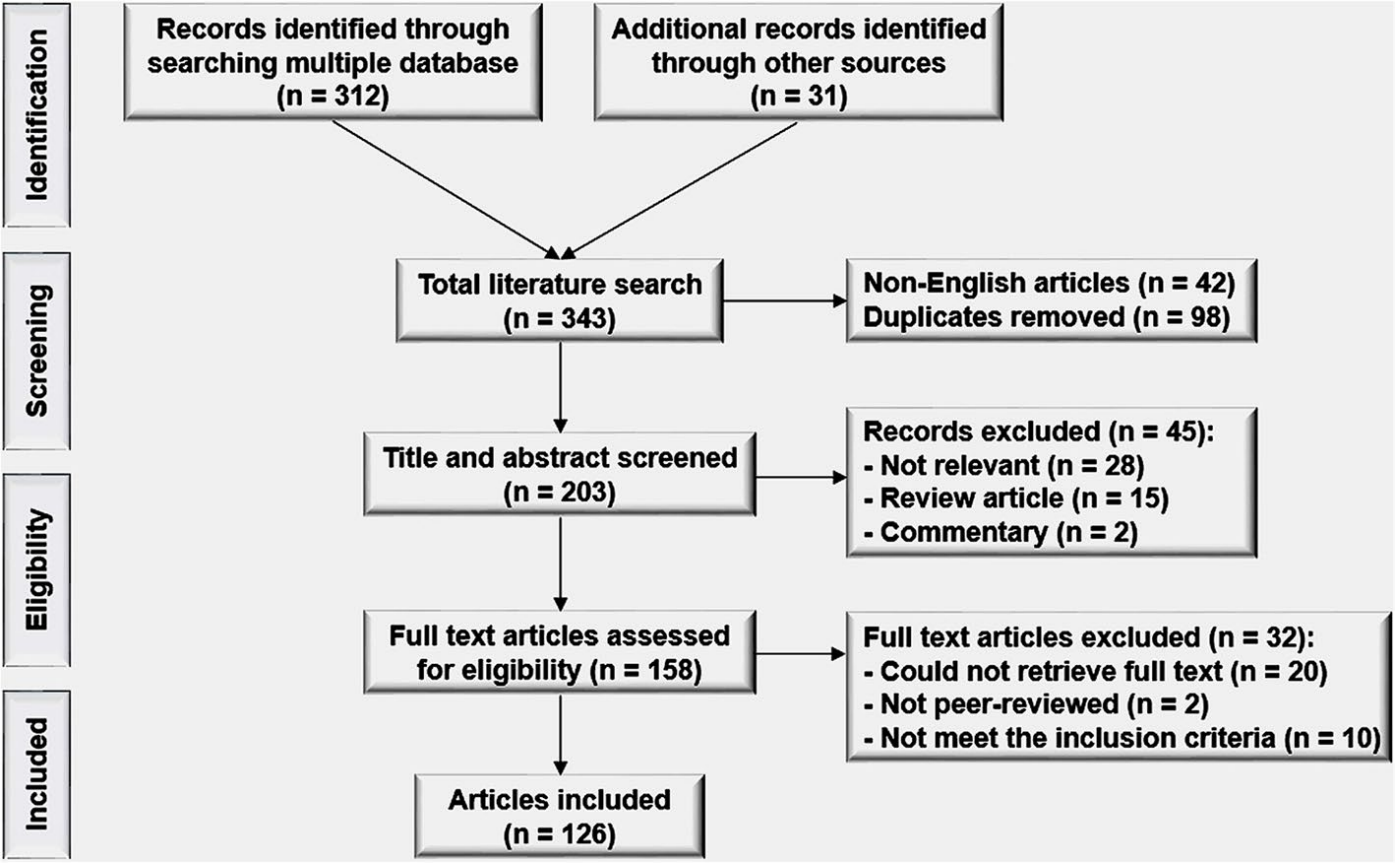
Screening and selection strategy of this review article. The literature searches produced 343 research articles, with 98 duplicates and 42 non-English articles, resulting in 203 articles being screened. After reviewing all article titles and abstracts, the full texts of 153 articles were further reviewed for eligibility. After removing articles that did not meet the inclusion criteria, 126 articles were eligible for inclusion in the study

Articles that did not describe the relevant contents were excluded after a preliminary check of titles and abstracts. In addition, review articles and commentary records were also excluded at this stage. After these screening steps, further exclusion proceeded after an assessment of the full text (Fig. 1).

### Data extraction and summary

We divided the included articles into four categories related to OG: “definition and symptoms”, “diagnosis with testing methods”, “galvanic corrosion” and “oral precancerous lesion with galvanism.” Every eligible article in these four categories was extracted by the author sequentially by using a custom-made data extraction sheet on Microsoft Excel.

## Results

### Searched results

Among 343 initially returned articles, 203 articles were excluded in the first screening step, with 98 duplicates and 42 non-English articles. After the next step, in which titles and abstracts were reviewed, 158 articles were left for the full-text review. Ultimately, there were 126 articles remaining that met the inclusion criteria (Fig. 1, Table 1).

#### Definition and symptoms of OG

**Fig. 2:**
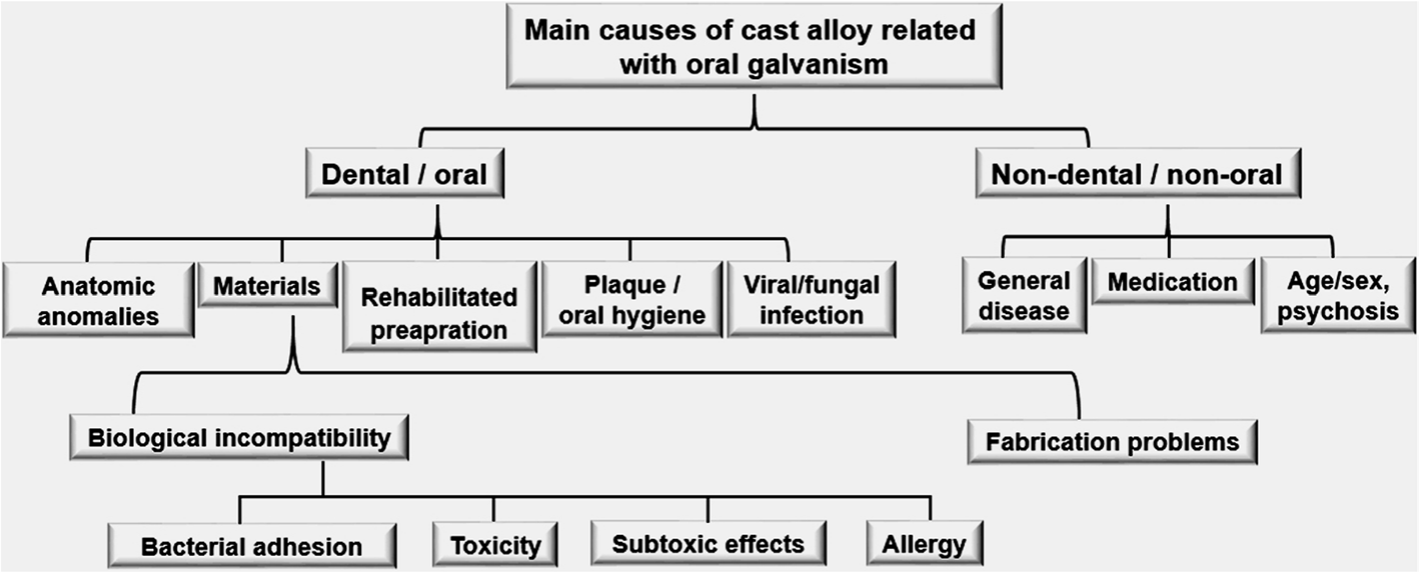
Table of the main causes of cast alloy related with oral galvanism could be divided as dental origins including anatomic anomalies, materials, rehabilitated preparation, dental plaque, and viral or fungal reaction or non-dental origins including general disease, medication, and others such as psychological backgrounds. Dental materials with biological incompatibility, such as bacterial adhesions, toxicity, subtoxic effects, and allergy, were the representative cause of oral galvanism

OG is the previous name given to direct intraoral current that is a continuous flow of current in the oral cavity through insulators or conductor, such as a wire and a vacuum-like ion beams, from high to low potential. This OG has variable size, but its flow direction is always the same according to its polarity. OG is also known as electro-galvanism due to its unidirectional current of electric charge and is generated in the saliva-filled oral environment in the presence of two or more dissimilar metals. The most common OG is related to dental amalgam restorations, from which are released metallic oxidized ions on the metal surface. The main causes of cast alloy related to OG can be divided into those with dental and oral origins, including anatomic anomalies, materials, rehabilitated preparation, dental plaque, viral or fungal reactions, and those with non-dental reasons, including general disease, medication, and others such as psychological background (Fig. 2). Regarding dental materials, biological incompatibility such as bacterial adhesion, toxicity, sub-toxic effects, and allergy is representative causes of OG.

**Fig. 3:**
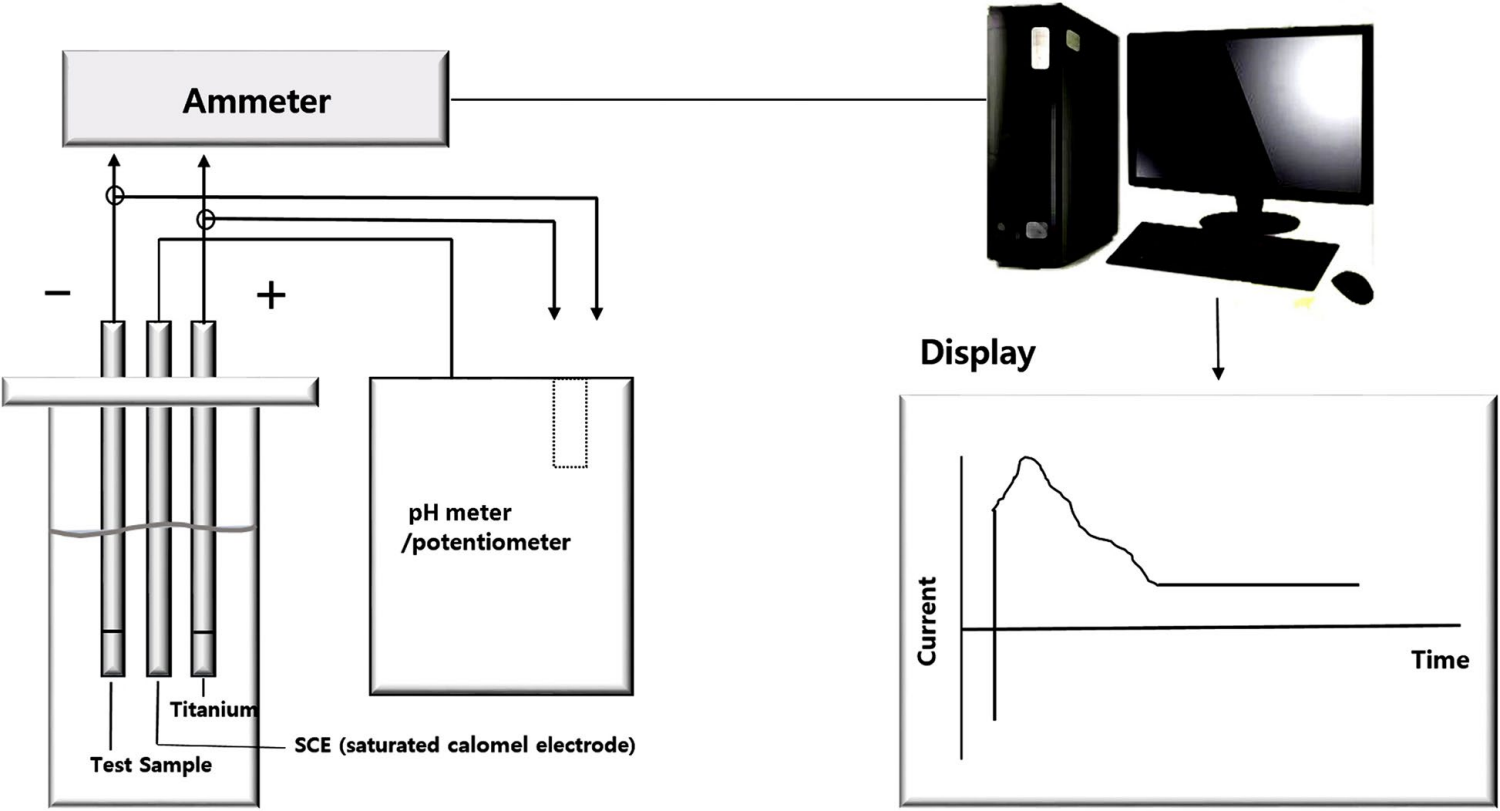
Schematic drawing of galvanic current measurement device having one electrode on the metallic prosthesis and another one for measurement of the potentials and currents

Galvanic current or a galvanic cell in the oral cavity is called a bimetallic cell with electrochemical differences from dissimilar intraoral metal alloys (Table 2). Ti and other metallic elements, such as cobalt (Co), nickel (Ni), gold (Au), palladium (Pd), mercury (Hg), iron (Fe), silver (Ag), and copper (Cu), have their own individual electrical potentials in the diverse intraoral situation. Thus, more than two different metals including titanium and its related implant prosthetic components might generate unexpected electromotive current [5, 8]. Unfortunately, this galvanic current cannot be measured easily due to its diverse electrical energy properties (Table 2), because intraoral Ti-related galvanic cells are often caused by different metal concentrations and bimetallic cells [14]. As with the galvanic concentration cells from dissolved oxygen in the chemically different electrolyte solution, this titanium ion can make an unexpected galvanic current in diverse intraoral saliva environments [15] (Fig. 3).

**Table 2:**
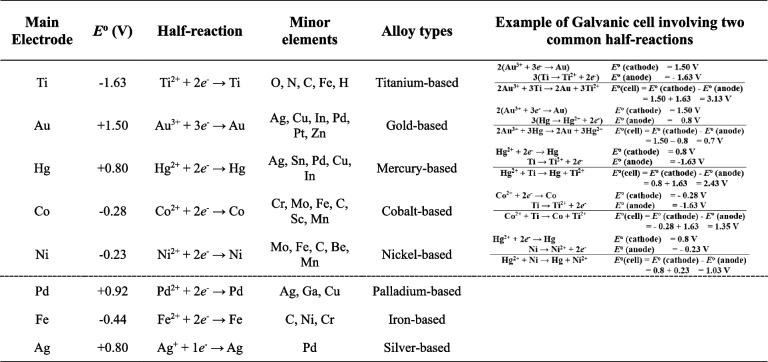
Main electrode in various alloy types and their standard electrode potential

*E*° standard electrode potential

The main symptoms of OG are an unpleasant burning sensation in the related teeth, intraoral metallic taste, disturbed smell, oral mucosal or tongue pain, and even atypical neuralgic pain. These atypical pains or discomfort can be expressed as emesis, vertigo, or continuous headache. Most of these symptoms occur after delicate changes in occlusal relationships of opposing teeth or in contact relationships of adjacent teeth after amalgam filling restorations or any metallic crown setting including implant crowns [16, 17]. An intraoral metallic taste is a persistent feeling with a vague sensation of smelling or tasting tinfoil taste after any metallic prosthesis delivery. A typical radiating unpleasant feeling is described, ranging from mild discomfort to a dull or worsening pain and ultimately as low-grade continuous pain.

Pain originating from OG has been noted both from an electronic circuit in the metallic restorations and from silver foil or spoon contact on biting [12, 15, 18]. More recently, these pains have originated from ion flow known as the battery effect in restorative dental works [12, 19, 20]. Severe pain might be referred to or projected to other body regions and experienced as headache, facial pain, or low back pain and also may be regarded as psychosomatic pain or discomfort. This psychosomatic pain of OG is a lower sensibility threshold and had a severe mental disease on a hidden psychosis of patient. Some of these patients could be found later to have a psychosomatic cause of pain [12, 21–24], and this pain might increase according to increased mental disturbances but also disappear easily after a recovery from psychosis.

#### Diagnosis with testing methods including immune markers

##### Clinical diagnosis and management of OG

Most patients experiencing OG visit the dentist initially and then a family doctor, internal medicine specialist, and otorhinolaryngologist complaining of intraoral pain or discomforts with unknown origins. Due to atypical signs or symptoms of OG, the initial diagnosis is somewhat difficult, but the first step is to collect anamnestic information including age, gender, main symptoms, onset and duration, general health, medical and dental history, and past or recent medications.

An intraoral examination is necessary for the exact identification of number and surface features of filling materials or metal restorations. This could be accompanied by routine radiograms including panoramic view and hematologic or urine analysis, and fungal contamination test may be carried out in partially edentulous or senior patients with saliva flow rates. However, the most important oral examination is the identification of metal restoration in the vicinity of surrounding mucosal inflammations, lichenoid changes of buccal mucosa, and metal-stained mucosa. The results of the visual exam are recorded as none, rare, slight, moderate, or severe, of which scales could be helpful for recording each patient’s own pain or discomfort level.

The patient’s main symptom is pain, which usually occurs after occlusal-related prosthesis fabrication or a recent filling treatment. If there are different materials or properties of metals related to the occlusal surface, the metal may be separated by rubber for the tentative clinical diagnose of OG, and any suspicious pain of galvanic currents might be relieved by the simple separations of the metallic prostheses [14, 17, 25]. In addition, even after a new prosthetic treatment is applied to an adjacent tooth, an initial clinical diagnosis is possible by separating the tooth in the same way. A barrier can be created with silver nitrate and composite resin, or other nonmetallic material can be used [14, 25]. If the patient’s pain symptoms disappear with simple separation, primary diagnosis and treatment may be possible by replacement with the same material-based prosthesis or filling materials or nonmetallic materials. A clinical OG diagnosis and replacement of related intraoral prosthetic materials to avoid contact between dissimilar metals can relieve pain and discomfort in sensitive patients. After removal of this galvanic cell, the patients can be educated regarding these situations and avoiding way for his/her further dental treatments.

For the psychiatric evaluation, it can be recommended to check several points such as lack of energy to cope with daily work or household duties, impaired quality of life, tiredness, anxiety, depression, inability to relax, sleeping disorder, and easy annoyances [26]. A full battery of psychological tests, such as BDI (Beck Depression Inventory), MMPI (Minnesota Multiphasic Personality Inventory), STAI (State-Trait Anxiety Inventory), and WAIS (Wechsler Adult Intelligence Scale), might be administered by a psychiatrist. The psychiatric interview is semi-structured, and the DSM-III (*Diagnostic and Statistical Manual of Mental Disorders*) or CMI (Cornell Medical Index) health questionnaire [27] can be used as basic criteria for a psychiatric diagnosis [12, 14, 28].

##### Laboratory diagnosis and intraoral testing of OG

All metal elements have their own electrode potential known as standard electrode (Table 2), and various dental metal alloys may influence intraoral galvanic effects due to electron potential differences. The protective passivation layers between the metal alloy and salivary electrolyte produce OG, and continuous abrasion and corrosion in this damaged interface worsen galvanic effects [29–31]. Most dental restorative alloys have the potential to cause intraoral hypersensitivity of the skin or intraoral mucosa. Common metal elements used in fabrication of dental crowns and bridges are Ni, chromium (Cr), Co, Pd, gold (Au), Ti, and Hg. Most noble metals such as Au or Pd are resistant to light or dry corrosion in hypersensitive skin when used in jewelry. However, even these noble metals might be corroded due to exposure to the moist environment of the oral cavity [22, 32–35]. However, it has been unknown which mechanisms are averse to metal alloys, nor pathognomic to innate immunity of oral cavity.

Heavy metals can be found in urine, feces, and blood tests. The most common metals tested are lead (Pb), Hg, arsenic (As), and cadmium (Cd), while less common metals include Cu, zinc (Zn), aluminum (Al), and thallium. Sometimes, hair analysis might be needed; for example, an Hg analysis of head hair was performed for investigation of metal exposure using cold-vapor atomic absorption spectrometry [36, 37]. Hair mineral analysis is used as a toxicological screening for metals including uranium (U), Pb, Hg, Cd, As, Al, and Ni.

For direct measurement of the sensibility threshold, electric stimulation using rising values of current can be performed by placing the negative electrode of an electronic pulp tester on the occlusal surface of each premolar or adjacent teeth. The indifferent electrode is placed on the occlusal surface of the premolar in the next oral quadrant, and the pulp tester is connected to voltage- and current-measuring instruments. The electrical current emitted by the tester is pulsated, the minimum current intensity evoking a sensation is recorded [38, 39], and the sensibility threshold is measured on the oral mucosa. To measure galvanic current between different dental alloys, the tongue, mucosa, and lips, several measurement devices were used, including an Odontologic 2000 device® (Embitron Co., Prague, Czech Republic) and an FfB Oralenergiemessgerat EM202® (FfB Co., Nürnberg, Germany) [40–42]. Every measurement device has a similar mechanism in which one electrode is placed on the metallic prosthesis, and the other is used for the measurements of the potentials and currents (Fig. 3).

For a better understanding of OG measurements, we evaluated international patents for the correlational research of OG with dental implant surface and prostheses. On the patent searching site https://patents.google.com/?q=Oral+galvanism&oq=Oral+galvanism, 211 search results were returned for “OG”, and 157 results were returned “dental implant galvanism” (https://patents.google.com/patent/EP1278479A1/en?oq=EP1278479A1, https://patents.google.com/patent/US8784104B2/en?oq=US8784104B2, https://patents.google.com/patent/RU2218078C1/en?oq=RU2218078C1, https://patents.google.com/patent/SE8604941D0/en?oq=SE8604941D0, https://patents.google.com/patent/RU2326620C1/en?oq=RU2326620C1, https://patents.google.com/patent/RU2462184C1/en?oq=RU2462184C1, https://patents.google.com/patent/RU2212185C1/en?oq=RU2212185C1) . Additional PCT searches on https://patentscope.wipo.int/search/en/search.jsf also returned 63 results under the keywords of OG and galvanic (https://patents.google.com/patent/US5725377A/en?oq=US5725377A, https://patents.google.com/patent/RO123606B1/en?oq=RO123606B1, https://patents.google.com/patent/KR100898726B1/en?oq=KR100898726B1, https://patents.google.com/patent/RU2018285C1/en?oq=RU2018285C1, https://patents.google.com/patent/CN103027757A/en?oq=CN103027757A, https://patentscope.wipo.int/search/en/detail.jsf?docId=SE227190350&_cid=P11-K7CNAM-76771-1, https://patentscope.wipo.int/search/en/detail.jsf?docId=RU29358799&_cid=P11-K7CNC5-77054-1, https://patentscope.wipo.int/search/en/detail.jsf?docId=RO233910277&_cid=P11-K7CNCZ-77146-1, https://patentscope.wipo.int/search/en/detail.jsf?docId=WO1997036551&_cid=P11-K7CNDF-77195-1) (Table 3). Different methods of galvanic current measurement in the oral cavity have been developed to observe the pathological effect of galvanic currents and voltage in the oral mucosa; nevertheless, correlative significance is still unknown [40, 43].

**Table 3:** International patent and PCT searching for oral galvanism

Title	Classifications	Publication date	Patent number (country)	Notes for galvanic
1. Equipment concerning depot of medicament in the mouth	A61C19/06 A61J7/0092	2003 January 23	EP1278479A1 (European patent office)	The slow-release device may contain one or several medicaments or a sacrificial anode
2. Method for diagnosing galvanosis in oral cavity	A61B5/05	2003 December 10	RU2218078C1 (Russia)	Potentials of metal structures relative to comparison chlorosilver electrode. Potential > ± 50 mV, galvanosis potential < ± 50 mV
3. Priority overcomer? 72R in the treatment of oral galvanism	A61K31/08 C07C43/10	1986 November 19	SE8604941D0 SE8604941L (Sweden)	Mixture of alkyl glycerols marketed as ECOMER (RTM) 3–6 capsules per day is taken, each containing 0.05 g of active material
4. Crown for differential diagnostics of allergic reaction etiology	A61C5/00	2008 June 20	RU2326620C1 (Russia)	The invention relates to medicine and is intended for differential diagnosis of the etiology of an allergic reaction
5. Method of diagnosing galvanosis	A61B5/05	2012 September 27	RU2462184C1 (Russia)	Measurement of value of bioelectromagnetic reactivity (BEMR) index. BEMR index, 0.047 to 0.94
6. Method for measuring galvanic processes in oral cavity	- A61B5/05 A61C13/38	2003 October 03	RU2212185C1 (Russia)	Measuring DC value flowing via metal structures available in oral cavity. The value is interpreted in terms of galvanic processes intensity in oral cavity
7. Osteoinductive screw implant	A61C8/00	2014 June 30	RO123606B1 (Romania)	Within the implant, a galvanic cell have the two poles connected to the implant extremities represented by the apical portion of the implant and the cover screw, separated from each other
8. Custom abutment structure of implant	A61C8/005	2009 May 20	KR100898726B1 (South Korea)	Producing a prosthesis (crown, porcelain) to normalize the psychological tooth arrangement and occlusal position
9. Micro electric field stimulation healing device for minitype dental implant	A61C8/00	2013 April 10	CN103027757A (China)	The invention discloses a micro electric field stimulation healing device for a minitype dental implant
10. Method and device for producing artificial denture	7A61C8/00 A 7A61C8/00	1999 January 19	RU02150250 (Russia)	Electric current of 5–50 mcA and concurrent exposure to magnetic field having inductance of 0.1–0.3 Tesla units

##### Immune markers of OG

OG could affect the immune defense reactions of the oral cavity and cause a range of different types of discomforts in the oral cavity. Most immunologic markers in the oral cavity are different levels in the human saliva with decreased levels of IgA1, IgA2, secretory IgA (sIgA), and lysozyme and an increased level of IgA against heat-shock protein 60 (anti-hsp60 IgA) [44–46]. Podzimek et al. [44] analyzed 397 oral discomfort patients and 30 patients with removal of electro-active dental materials and demonstrated the importance of measuring GC in patients having oral discomfort symptoms, such as inflammatory lichen planus on the tongue and buccal mucosa, gingival metal pigmentations, prosthetic corrosion, and metallic taste. Levels of IgA1, IgA2, sIgA, and lysozyme were increased, with decreased levels of anti-hsp60 IgA after removal of prosthetics, and clinical symptoms were also decreased in 70% of patients. This author described that oral prosthesis with more than 5 μA GC had an electro-active trend and recommended that at currents greater than 10 μA GC, the oral prosthesis be removed from the patient [44].

In another whole saliva study, proteins, Na, Cl, and P were significantly higher, and Ca, Mg, and IgA concentrations were lower, in OG patients; these proteins, lysozyme, and Ca concentrations were changed in the parotid saliva as well. However, pH did not change according to galvanic current differences or galvanic symptoms [41]. A decrease of saliva pH may affect local changes in biofilm contacting the metallic prostheses rather than galvanic current itself, followed by passivation or activation of the intraoral electric currents in the surfaces of metallic restorations.

Compared with B-lymphocyte responses, proliferation of T lymphocytes was significantly decreased by galvanic current and voltage changes. This situation could be prolonged by metal ion release in the oral cavity, which was also compatible with lymphocyte potential changes such as K^+^ and Ca^2+^ conduction block [47]. Functional K^+^ channels are necessary for T-lymphocyte proliferation [48, 49], and values of galvanic currents and voltage affect potentials of K^+^ channels, leading to T-lymphocyte malfunction [44]. The pathologic intensity values of galvanic current and voltage ranged from 3 to 10 μA and from 80 to 200 mV [50], depending on the surface molecules including CD 3, 19, 11a/18, 3/69, 3/95, 19/69, and 19/95 from lymphocytes. Additionally, pathological threshold values of 5 μA for galvanic currents and 100 mV for galvanic voltage were reported [44].

### Galvanic corrosion

**Fig. 4:**
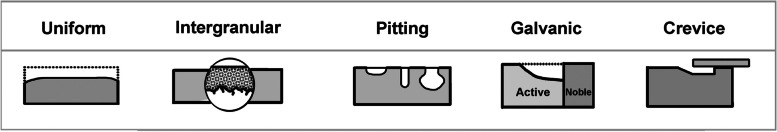
Schematic drawing of different metallic corrosion types by shape, including uniform, intergranular, pitting, galvanic, and crevice corrosion

Generally, metal corrosion can be defined as a phenomenon in which a metal material undergoes a chemical or electrochemical reaction with the surrounding environment, deteriorating the material properties or shortening its effective useful life. Metal corrosion can be classified as high- or low-temperature corrosion [47, 48]. Atmospheric, seawater, soil, and microbial corrosion are divided according to the environmental change. In addition, shape differences such as uniform, intergranular, pitting, crevice, and galvanic corrosion are different corrosion types, with selective leaching and hydrogen damage types as well (Fig. 4).

Incidence of metallic corrosion increases with increasing temperature and Cl and decreasing Cr and Mo contents of the metal [10, 49]. Intergranular corrosion, or intergranular attack, may occur where the boundaries of crystallites of the material are more susceptible to corrosion than their insides. Pitting corrosion is similar to crevice corrosion (CC), which is brought about by local damage to the passive film, with chlorides playing a significant role [48–50]. The hydrolysis of the released corrosion products decreases the pH value of the electrolyte in the pit or crevice, accelerating corrosion [50]. CC, a localized attack on a metal surface, may occur at the gap or crevice between two joining surfaces. These gaps or crevices can be formed between two metals or between a metal and a nonmetallic material. CC refers to corrosion that occurs in confined spaces that are difficult to clean or difficult to access, commonly referred to as crevices. Among various intraoral crevices, a gap between a prosthesis and the remaining tooth, or a crevice between the boundary of a prosthesis and other fillings, can be considered. In these intraoral crevices, oxygen is insufficient, or the movement of oxygen is restricted, so that hydrolysis corrosion products are generated in the crevices. This results in local decay of the passive state due to a decrease in pH, which is particularly common in stainless steels when chlorides are involved. Decayed pits, scratches on alloys due to abrasion or insufficient finishing in dental laboratories, and interdental spaces or tight spaces between different parts of a restorative structure can be considered intraoral crevices, and these CCs include special microenvironments in the oral cavity. Diverse dental restorations, including dental implants, are often fabricated to extend below the gingival margin into the gingival fissure, and physiological fissures or crevices also occur.

Intraoral electrochemical corrosion is also known broadly as OG. However, the exact terminology with GC is defined as an ionic release from intraoral metallic restorations, which can cause local or systemic pathological problems associated with OG [14, 15, 51]. GC, or bimetallic corrosion, is the contact corrosion between two dissimilar metals or alloys under the circumstances of electrolyte. This electrolyte makes a driving force named electrochemical potential between two dissimilar alloys, such as body fluids or saliva. Several metal ions released from dental alloys, albeit in small amounts, have short- or long-term effects in the oral cavity through electrochemical corrosion and wear [40, 41, 52]. In addition, the concentration of metal ions in saliva rapidly increases [40, 41].

**Fig. 5:**
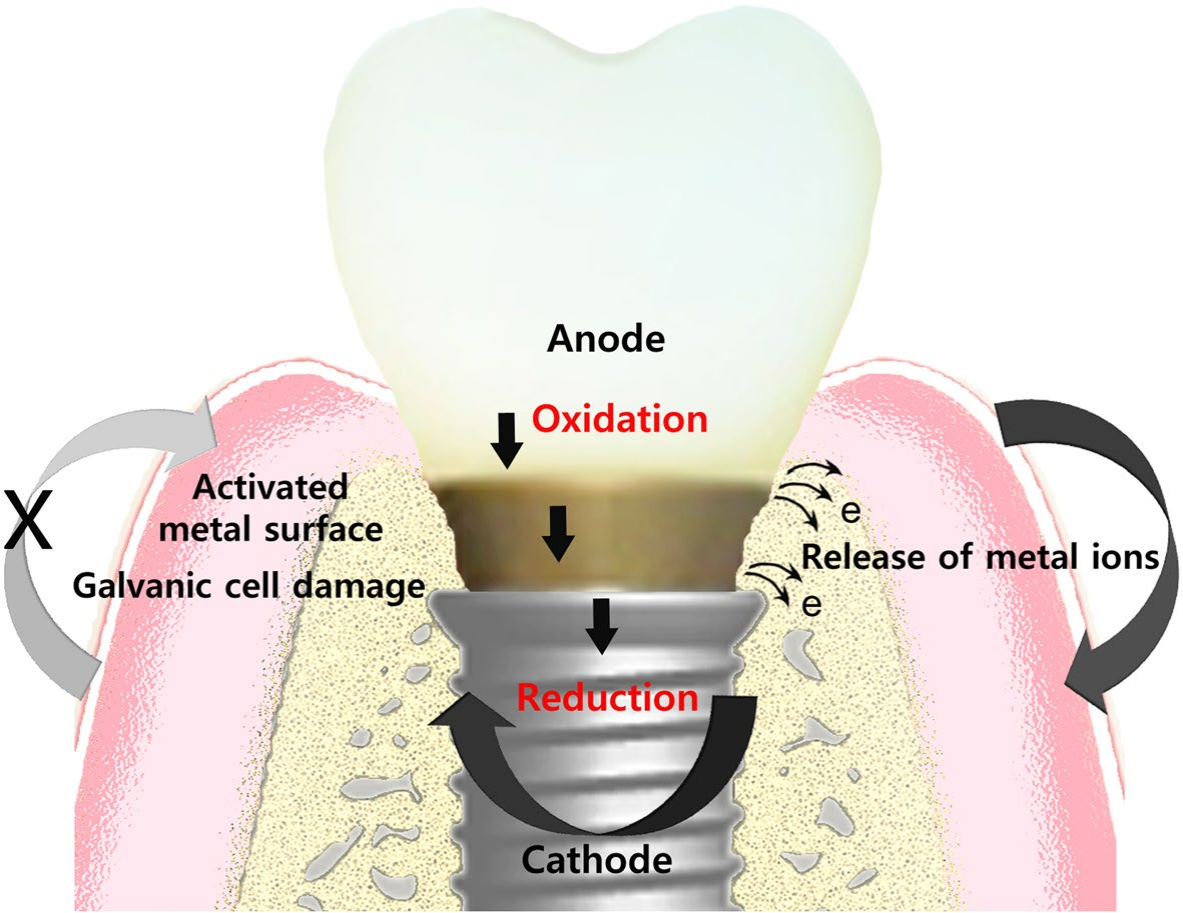
Galvanic cell formation near the junction between the implant abutment and fixture showing an inevitable chemical reaction between the titanium fixture, titanium abutment, and its accompanied implant crown or other prosthesis, known as implant corrosion or implant galvanic cell damage

**Fig. 6:**
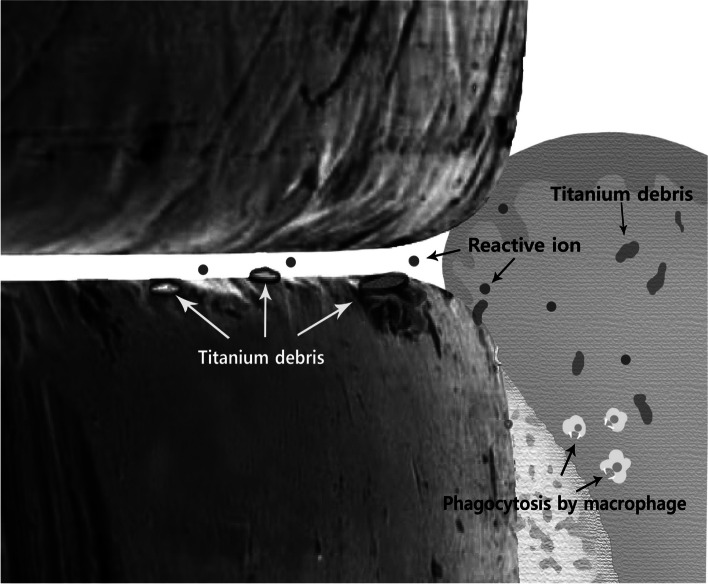
Schematic representation of Ti debris and reactive ions related to galvanic corrosion in the implant-attached gingival connection. Titanium debris in the implant connection part corroded by reactive ions and phagocytosis by macrophages in the junctional epithelium

Clinically, GC occurs in a variety of intraoral situations, such as when dental alloys are in direct contact with each other, amalgam fillings are placed right next to a crown, or when opposing metal restorations that are occluding cause mutual interference during jaw functions such as mastication and swallowing. Intraoral GC also occurs as an inevitable chemical reaction between the oral environment and dental implants, which have an essential titanium fixture, titanium abutment and its accompanied implant crown, or other prosthesis. At least two or three different interfacial surfaces between them could release diverse metal ions to activate metal surfaces of each component, resulting in galvanic cell damage near the implant abutment-fixture junctional area (Figs. 5 and 6). This inevitable chemical reaction of dental implant connections is known as intraoral implant corrosion or implant galvanic cell damage.

Dental alloys are classified into high-grade noble, precious or noble, non-precious, and Ti alloys. High-grade noble metals or Au-based alloys include Au, platinum (Pt), and Pd. Noble metals that are reasonably priced and biocompatible include Pd-based alloys and Au, Ag, Cu, and gallium (Ga). Representative examples of non-precious metals are stainless steel and nickel-titanium (Ni–Ti) alloys mainly used in orthodontic appliances and nickel-chrome (Ni–Cr) and chrome-cobalt (Cr-Co) alloys mainly used in dentures and fixed prostheses. On the other hand, Ti and Ti alloys known for their biocompatibility have been used mainly for dental implant fixtures and superstructures and are widely believed to be biocompatible.

Currently, all dental implant fixtures are made of Ti with a purity of 99wt% or more, and more precisely, they are made of an alloy such as Ti6Al4V containing 6wt% Al and 4wt% V. Most of the abutments, which are superstructures for implant prostheses, are also made of Ti or Ti alloys, and these Ti and Ti alloys show an oxidation reaction immediately when exposed to air. This oxide layer forms a passive layer with a thickness of 10–20 nm to prevent Ti metal from being corroded. However, it is easily affected by mechanical force or exposure to fluoride and other causes of corrosion [47, 50], which could lead to GC. A patch test is often used as an allergy test for metal, but metal allergy to oral diseases is difficult to identify as an allergen by using a skin patch test [31, 46]. In particular, it is known that Ti does not penetrate through the skin, which is considered to be because it immediately forms an early oxidation layer [17, 48]. In particular, TiO_2_ is known to easily penetrate the oral mucosa [49, 50]. Ti-related allergic reactions have been noted in various in vitro studies [7, 49, 50] but have been rarely known clinically due to the difficulty of confirmation, and the Ti patch test is also unreliable. After dental implant placement, Ti-induced lymphocyte proliferation increased in half of 56 patients with various systemic health problems, and it has also been reported that removal of the implant showed significant health improvement [32]. In in vitro tests such as lymphocyte proliferation or transformation tests (LPT/LTT), sensitization to Ti has been frequently reported at 4.2–42%, but it is difficult to relate the results of these in vitro tests clinically [47, 53].

The galvanic cells caused by the release of metal ions can have various oral and systemic effects, and it is reported that the higher the value of the measured galvanic current, the more frequent the inflammatory symptoms of the oral mucosa [54, 55]. Crevice corrosion in the oral cavity is activated not only in an acidic environment but also under the influence of lipopolysaccharides [54–56], and acidic foods such as soft drinks and fruits and oral infections lower the salivary pH to 2–3 levels [49]. Conflicting data have been presented for the toothpaste commonly used to prevent oral cavities [57]. Toothpaste contains fluoride, which can cause abrasion of metal surfaces in the oral cavity and can have a detrimental effect on high concentrations of Ti [58]. Studies of pure Ti and Ti alloys immersed in fluoride and bleaching agents have shown unexpected corrosion [32, 59], and this mechanical wear results in a more severe and rapid corrosion process between two or three different metal alloys.

GC can always occur because the various alloys in the oral cavity are all different in composition, and even if the alloys do not come into direct contact, they are connected to each other through saliva in the oral cavity. Cases involving multiple metal alloys and Ti alloys in the oral cavity are increasingly common.

### Oral precancerous lesion with galvanism

**Fig. 7:**
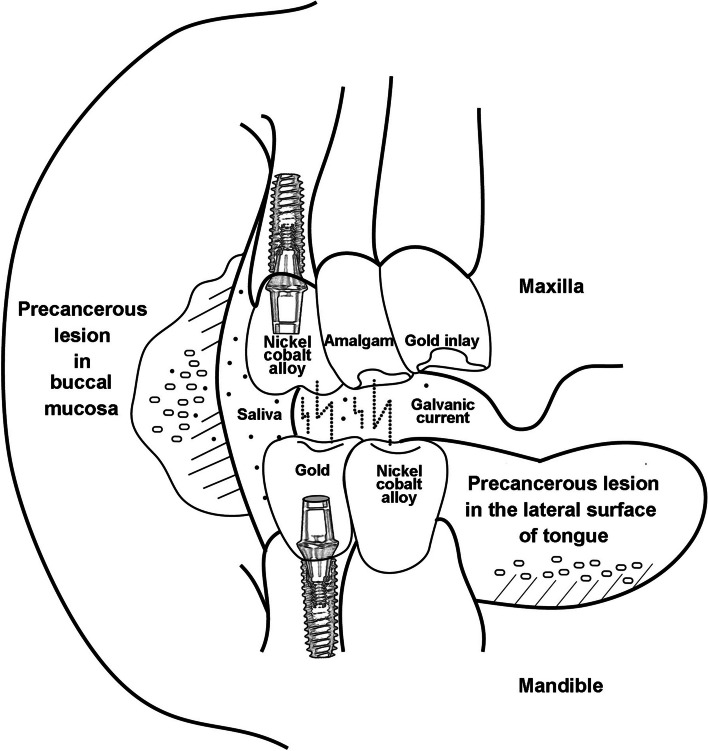
Drawings of chronic galvanic irritation in the oral cavity. Erosive or erythematous precancerous mucosal lesion might be caused by ionic-free radicals and accumulation of these ionic concentrations in the abundant salivary buffer adjacent to buccal mucosa or lateral surface of the tongue

Unlike previously described OG-related symptoms, several mucosal lesions, including oral precancerous and cancer lesions, represent more severe disease prognoses and even threats to life. Before a patient’s unusual discomfort or complaint begins, OG-induced mucosal lesions should be identified in their early stage and prevented. The presence of intraoral dissimilar metal alloy restorations is not related to an increased incidence of oral mucosal lesions, but any form of GC or galvanic current could make the associated mucosal disease in the oral cavity [39]. Toxic effects of dissimilar metal ions on the adjacent mucosal tissues could induce unexpected situations, associated with OG-related disease. Most oral mucosal atypical gray-white lines occurred as lace-like elevated lines bilaterally in the buccal mucosa, named Wickham’s striae, and in the lateral surface of the tongue. From these, atypical but normal mucosa could be changed as erosive or erythematous patterns with patient’s symptoms, named leukoedema, leukoplakia, or OLP [60]. Although OG usually do not induce ulcerative mucosal lesions, minor chronic galvanic irritation might affect these erosive or erythematous precancerous mucosal lesions by producing ionic-free radicals and accumulating these ionic concentrations in the abundant salivary buffer, especially adjacent to buccal mucosa or lateral surface of tongue and especially to implant-related sulcus and junctional epithelium (Fig. 7).

Sensitization to metal restorations is suspicious in this stage for easy pathologic processing under the amalgam, Au, Pd, and even titanium [12, 13, 18, 31, 61]. If there is an implant restoration with abutment connection involving dissimilar materials, these atypical mucosal lesions could be widespread due to its more complexed dissimilar metal elements (Fig. 7). Galvanic currents could also spread through the oral mucosal surface and adjacent tongue surface and increase the local galvanic symptoms by increasing ionic contents in the saliva. More than 5-μA galvanic current has been reported as the threshold of intraoral inflammation in the buccal mucosa and tongue surface combined with other symptoms including glossodynia, neuralgia, stomatodynia, and hyperemia of tooth pulp [14, 15, 39, 40]. A representative mucosal pathologic study induced by electronic current showed that the mean value of the galvanic current was 13.4 ± 10.3 μA, compared to less than 5 μA in the normal group among 159 examined OG patients [46]. In addition, higher values of galvanic currents, 22.8 ± 12.5 μA, were also found in patients having oral mucosal changes. Thus, OG generated by more than two adjacent metal prostheses could aggravate these galvanic current flows [8].

Leukoedema, the abundant edematous keratinocytes, is known to have a good amount of cytoplasmic fluid instead of polymerized keratin filaments. This is responsible for the change of extracellular osmotic pressure elicited by increased ionic concentration of galvanic current; thereby, the superficial layer keratinocytes of leukoedema rapidly lose their cytoplasmic fluid and are subsequently shrunken and exfoliated [11, 12]. The leukoedema epithelium can be rapidly ulcerated on the galvanic current strength generated from occluding metallic restorations. As a result, electrical actions from prosthetic metal materials of crowns and/or bridges should be considered an important etiologic factor of OSCC of the buccal mucosa and tongue (Fig. 7). Practically, after modifying an intraoral prosthesis or making a new one, several patients might have been diagnosed as the initial stage of OSCC on the lateral tongue or buccal mucosa [13]. Thus, the prosthesis may act as a type of weak battery in the oral cavity, and accordingly, electrical current battery may result in OG irritation to the adjacent tissues.

Galvanic phenomena in the oral cavity can increase the proliferation of leukoplakia cells, induce apoptosis, and stimulate some morphological changes in OSCC, which is called peri-implant oral malignancy (PIOM). Galvanism, which affects ornithine decarboxylase, is upregulated in several cancers because Na + K + -ATPase acts as an ion transporter [11, 12]. Galvanometer measurement, local galvanic cell action, and polarization effects were already known main factors of tongue SCC from a 1934 report [8, 13]. These three comments have been the essential factors used for the evaluation OG levels and the exact diagnosis criteria in OG-suspicious OSCC patients.

Various immune cells present in the oral mucosa respond to various antigens such as lipopolysaccharide in a tolerogenic manner [45]. This explains why patients allergic to nickel have relatively few oral diseases, even when exposed to multiple stimuli from various pathogenic bacteria and exogenous antigens while wearing nickel-containing orthodontic appliances intraorally. It is known that patients with skin allergies caused by metals such as nickel and cobalt also have immune tolerance when orthodontic appliances are used in the oral cavity [33, 62]. As mentioned earlier, the mobile tongue mucosa is a good conductor, and saliva is composed of various electrolytes. When the tongue is moved, different metals pass through an external conductor circuit composed of new electrolytes to form metal-to-metal currents. Therefore, an implant-supported prosthesis placed in contact with the lateral border of the tongue conductor immersed in salivary flow may form an unexpected galvanic battery in the oral cavity (Fig. 7).

In vitro studies demonstrated that electromagnetic fields could induce intracellular changes in OSCC cells and initiate apoptosis in oral mucosal cancer cells. [63]. In vitro studies of low-frequency electromagnetic fields revealed the physiological endpoints of many different sensitive cells, and effects on DNA, RNA and protein synthesis, cell proliferation, cation flux and binding, immune response, and membrane signaling have been reported. This occurred mostly with short exposure of the cells to frequencies below 100 Hz and low electromagnetic intensities [64]. Additionally, long-term exposure to electromagnetic fields can increase the risk of developing tumors by sustainably increasing levels of free radicals and increasing the incidence of DNA damage [46]. Electric voltages in dental metal alloys used for dentures, restorative materials, and orthodontic devices can occur up to 950 mV between different dental alloys, and 80–200 mV of OG could induce subcellular changes of MSK-LEUK1, an oral leukoplakia cell line, in the closely simulated morphological features of PIOM having circumstances in vitro [24].

## Discussion

Intraoral electromechanical reaction between dissimilar metals has been a concern when a patient complains of an unpleasant taste or pain. In recent times, dental implant management has become more prevalent, leading to an increase in oral galvanism-related symptoms. Many patients can be treated by adjusting their occlusal contacts or by replacing metal crowns. The comparative high currents of OG may affect mucosal inflammation and even precancerous lichenoid lesions, especially on the buccal mucosa and lateral surface of the tongue related to implant restorations. Low currents of OG could also induce symptomless metallic pigmentation around an implant restoration and might worsen if not treated.

When the threshold of sensitivity is lowered, the patient becomes more sensitive to internal and external stimuli that are generally not felt or recognized. The increased sensitivity of these patients may be related to personal mental disorders and to extrinsic stimuli like intraoral candidiasis or other allergens [12, 25]. These complex-sensitive situations might be diagnosed, not from the pain-reducing medications but from intraoral galvanic current changes, which might be checked in the immune system and in saliva composition. Thus, treatment must be guided by these practical findings, even in confusing situations, with the goal of raising the sensitivity threshold through the assessment and encouragement of any psychological disorders [12, 27, 32, 58]. Intraoral metals or dental implants are not physiological components of the human body such as alveolar bone or gingival tissue. Various oral discomforts may occur in association with symptoms such as allergies and metal hypersensitivity after implant restoration [31, 39], and particularly, undesirable side effects may occur in sensitive patients [22, 39].

Several types of psychological testing or interviews also could be essential for OG in the second or third stage of management during a treatment course. Implant crown metal changes with occlusal adjustment of implant management should be done initially, with periodic checkups combined with OG patient hormonal changes or other social environmental factors. However, these detailed examinations are very difficult to incorporate into our daily routine clinical procedures. Saliva flow rate with its individual composition cannot be measured easily, and it is impossible to measure each patient’s mental stress, which has remained the weakest point of OG diagnosis.

Although the various and complex corrosion processes in the oral cavity are difficult to quantify, it is recognized that various metal ions, such as Ni, are released from various dental alloys, and that some metal ions, such as Au and Pd, are absorbed into the body. Because the compositions of various dental alloys are different in the oral cavity, GC can always occur, and this is because there is a medium called saliva even if the alloy is not in direct contact.

As dental implants become more common, various oral cavity environments including a certain level of infection in the oral cavity, changes in fluoride concentration due to the use of fluoride-containing toothpaste, and various elements and free radicals, are expected to emit other implant-related metal ions. There is a salivary environment in the oral cavity containing all. Although it may not be believed, it is certain that various metal alloys and Ti alloys will continue to change the oral environment in the near future.

## Conclusions

Patients with OG have high oral energy and current, and although this phenomenon may be due to the patient’s mental illness, OG due to amalgam or mercury occurs. Therefore, it is also evident that the difference in electron potential caused by different elemental components such as titanium alloy and pure titanium, which are essential for manufacturing the implant fixture and the abutment, and chrome and nickel, which are essential for manufacturing the upper crown, causes OG. GC is a continuous process that increases with time in the oral cavity, especially in the presence of crevices or pitting corrosion. Although the actual symptoms may differ according to individual differences and sensitivity of patients, there is no standardized method for diagnosing intraoral OG by implants. It should be noted that these differences can occur biologically and chemically.

Above all, since the oral cavity is equipped with an environment in which electric current can be transmitted easily due to saliva, it is imperative that clinicians review the systemic and local effects of salivation. It may be an infrequent situation, but it is worth noting that there are increasing numbers of cases in which various metal alloys and Ti alloys are present in the oral cavity.

## Data Availability

This study was conducted as a retrospective research method, and raw data can be provided at the request of the journal.

## Abbreviations

OG: Oral galvanism
Ti: Titanium
GC: Galvanic corrosion
OSCC: Oral squamous cell carcinoma
Co: Cobalt
Ni: Nickel
Au: Gold
Pd: Palladium
Hg: Mercury
Fe: Iron
Ag: Silver
Cu: Copper
Cr: Chromium
Au: Gold
Pb: Lead
As: Arsenic
Cd: Cadmium
Zn: Zinc
Al: Aluminum
U: Uranium
sIgA: Secretory IgA
CC: Crevice corrosion
Pt: Platinum
Ga: Gallium
Ni-Ti: Nickel-titanium
Ni-Cr: Nickel-chrome
Cr-Co: Chrome-cobalt
OLP: Oral lichen planus
PIOM: Peri-implant oral malignancy
